# Preliminary Investigation of the Dissolution Behavior, Cytocompatibility, Effects of Fibrinogen Conformation and Platelet Adhesion for Radiopaque Embolic Particles

**DOI:** 10.3390/jfb4030089

**Published:** 2013-07-10

**Authors:** Sharon Kehoe, Marie-Laurence Tremblay, Aisling Coughlan, Mark R. Towler, Jan K. Rainey, Robert J. Abraham, Daniel Boyd

**Affiliations:** 1 Department of Applied Oral Sciences, Dalhousie University, PO Box 15000, Halifax, NS B3H 4R2, Canada; 2 Department of Biochemistry & Molecular Biology, Dalhousie University, NS, B3H 4R2, Canada; E-Mails: mltremblay@dal.ca (M-L.T.); jan.rainey@dal.ca (J.K.R.); 3 Inamori School of Engineering, Alfred University, Alfred, NY 14802, USA; E-Mail: coughlan@alfred.edu; 4 Faculty of Engineering & Architectural Science, Ryerson University, 350 Victoria Street, Toronto, ON, M5B 2K3, Canada; E-mail: mtowler@ryerson.ca; 5 Department of Chemistry, Dalhousie University, NS B3H 4R2, Canada; 6 Department of Diagnostic Imaging and Interventional Radiology, QE II Health Sciences Centre, Dalhousie University, Halifax, NS B3H 4R2, Canada; E-Mail: bobjabraham@gmail.com; 7 Room 3316, Halifax Infirmary Site 1796 Summer St., Halifax, NS, B3H 3A7, Canada; 8 School of Biomedical Engineering, Dalhousie University, PO Box 15000, Halifax, NS B3H 4R2, Canada

**Keywords:** embolic particle, dissolution, cytotoxicity, fibrinogen conformation, platelet adhesion

## Abstract

Experimental embolic particles based on a novel zinc-silicate glass system have been biologically evaluated for potential consideration in transcatheter arterial embolization procedures. In addition to controlling the cytotoxicity and haemocompatibility for such embolic particles, its glass structure may mediate specific responses *via* dissolution in the physiological environment. In a 120 h *in-vitro* dissolution study, ion release levels for silicon (Si^4+^), sodium (Na^+^), calcium (Ca^2+^), zinc (Zn^2+^), titanium (Ti^4+^), lanthanum (La^3+^), strontium (Sr^2+^), and magnesium (Mg^2+^), were found to range from 0.04 to 5.41 ppm, 0.27–2.28 ppm, 2.32–8.47 ppm, 0.16–0.20 ppm, 0.12–2.15 ppm, 0.16–0.49 ppm and 0.01–0.12 ppm, respectively for the series of glass compositions evaluated. Initial release of Zn^2+^ (1.93–10.40 ppm) was only evident after 120 h. All compositions showed levels of cell viabilities ranging from 61.31 ± 4.33% to 153.7 ± 1.25% at 25%–100% serial extract dilutions. The conformational state of fibrinogen, known to induce thrombi, indicated that no changes were induced with respect of the materials dissolution by-products. Furthermore, the best-in-class experimental composition showed equivalency to contour PVA in terms of inducing platelet adhesion. The data generated here provides requisite evidence to continue to *in-vivo* pre-clinical evaluation using the best-in-class experimental composition evaluated.

## 1. Introduction

Embolization has emerged as a highly effective technique in the treatment of a wide variety of pathologies, including arteriovenous malformations [1] and hypervascularized tumors (such as hepatocellular carcinoma [2], and uterine leiomyoma [3,4]). Due to the availability of multiple particle size distributions and a comprehensive history of clinical safety and efficacy, polyvinyl alcohol (PVA) and tris-acryl gelatin particles are extensively used for embolization procedures [5,6]. These agents however have limitations, most significantly, the particles themselves are radiolucent. Consequently, clinicians are not able to directly monitor the placement of embolic particles radiographically, resulting in undetected cases of reflux with “*non-target embolization*” [7] and “*through embolization*” [5]. Dispersing the particles in radiographic contrast media ameliorates this issue. However, exposure to contrast media is commonly linked to contrast-induced nephropathy (CIN); the third most common cause of hospital acquired renal failure [8,9,10]. 

Recently, focus has shifted towards developing new radiopaque polymeric agents loaded with iodine [11,12], iohexol [13], iopamidol [14], and lipiodol [15]. However, all of these radiopaque embolic agents still rely on the incorporation of contrast media to enhance its visibility under X-ray. While barium sulfate [16] and tantalum [17] loaded polymeric embolic microspheres were previously developed by the same group, these particles are not fully loaded with these radiopaque additives and therefore remain only partially visible under X-ray. As such, there still exists a recognized clinical need for fully radiopaque embolic particles that minimize the requirement for contrast media.

The authors have recently investigated a series of new material compositions, which provide for intrinsically radiopaque embolic particles [18,19] and, as such these new agents may facilitate reduced use of contrast media, and permit direct visualization of the embolic agents during clinical delivery; features which may significantly improve patient safety. Most recently, the authors have reported a preliminary investigation for an implantation study demonstrating the safety and efficacy for this glass particulate in a short-term (21-day) study alongside intracutaneous irritation testing performed using New Zealand White (NZW) rabbits versus PVA [18]. 

In the initial studies, fundamental material properties were correlated against compositional variations to establish mathematical formulae based on mixture designs [19]. Having established their composition-structure-property relationships, it is now necessary to begin an examination of whether the materials can provide for an appropriate materials and host response with respect to embolization. In this regard, the present work contains a number of synergistic objectives, including:

1. Examining materials response (generation of dissolution by-products) after short-term exposure to a simulated physiological environment;
2. Evaluating the cytotoxicity of serial extract dilutions (elution assay), and substantiate the identification of a preferable composition for pre-clinical evaluation;
3. Examining the likely host response, by examining the state of fibrinogen (Fg) conformation with regards the dissolution by-products and the haemocompatbility (*i.e.*, platelet adhesion) of the materials particle surface.

To fulfill these objectives, this paper describes a short-term time-release dissolution study to quantify the release levels for all possible dissolution by-products derived from the new embolic agents: Si^4+^, Na^+^, Ca^2+^, Zn^2+^, Ti^4+^, La^3+^, Sr^2+^, Mg^2+^ and Zn^2+^; such an analysis is a critical component in the biological evaluation of a new medical device [20,21]. Following this analysis, the same extracts were subjected to MTT-assaying with NIH-3T3 fibroblasts at serial dilutions of 25%, 50% and 100%. Furthermore, undiluted extracts were used to examine if the ionic dissolution by-products might induce unwanted changes in Fg conformation, leading to site-specific thrombus formation [22] on the surface of the dissolution by-products as opposed to the surface of the implanted particle itself (*as required to ultimately limit blood supply to the intended target area*). In respect of the latter, Fg when exposed to biomaterial surfaces is widely accepted to be an important mediator in platelet adhesion [23]. Fg molecules have approximate dimensions of 4.5 × 47 nm [24]. Fg is the third primary plasma component [25,26], being one of the most relevant proteins investigated with respect to: (i) blood coagulation; (ii) facilitation of adhesion; and (iii) aggregation of platelets, all of which are important properties in the process of thrombosis. Specifically, Fg was chosen for this study due to its key role in the blood coagulation process. Although the importance of Fg conformation has been shown for a vast range of material surfaces, there have been limited studies that examine the role of Fg conformation with respect to surfaces of dissolution by-products using circular dichroism spectroscopy [27]. This study addresses this anomaly. Finally, this study uses human blood for the *in vitro* assessment of the materials haemocompatibility determined through lactate dehydrogenase (LDH) activity with platelets. As such, this paper provides an initial evaluation of material and host responses for novel embolic particles that have already demonstrated high levels of radiopacity [18], for use in peripheral vascular embolization.

## 2. Results and Discussion

In our previous study [25], we characterized the experimental embolic particles physico-chemical properties using X-ray diffraction (XRD) analysis, ^29^Si magic angle spinning nuclear magnetic resonance (MAS NMR) and helium pycnometry. The same series of embolic particles underwent further characterization in this study to assess their early material and host responses.

### 2.1. Material Synthesis

Eight embolic particle compositions according to Table 1, previously defined [19] as suitable for synthesis *via* the melt-quench technique were further evaluated herein (namely *ORP1, 2, 3, 5, 6, 7, 9, and ORP 11*)*.*

**Table 1:** Compositions of embolic particulates (values expressed as mol. fraction. Note CaO, Na_2_O, MgO and SrO are present in each composition at equimolar concentrations of 0.035 mol. fraction).

Embolic designation	Variable concentrations			
	ZnO	La_2_O_3_	SiO_2_	TiO_2_
ORP1	0.137	0.137	0.553	0.033
ORP2	0.240	0.000	0.570	0.050
ORP3	0.213	0.068	0.537	0.042
ORP5	0.188	0.068	0.562	0.042
ORP6	0.068	0.188	0.562	0.042
ORP7	0.213	0.068	0.562	0.017
ORP9	0.290	0.000	0.520	0.050
ORP11	0.290	0.000	0.570	0.000

### 2.2. Material Morphology and Particle Size Distribution

**Figure 1:**
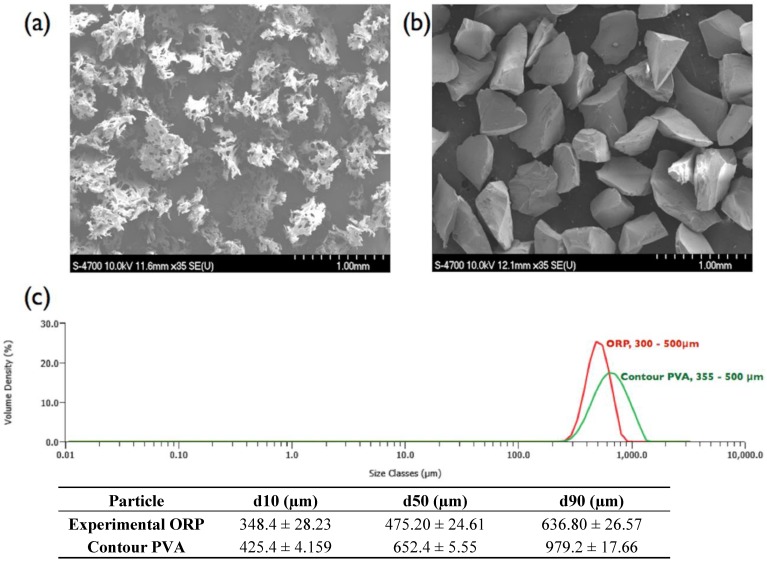
(**a**) SEM of commercial irregular contour PVA; (**b**) experimental ORP particles (representative of all ORP compositions); and (**c**) corresponding particle size distribution analysis. *Scale bar = 1.0 mm.*

Figure 1a,b compares the morphology of the experimental embolic particles (PSD: 300–500 µm) reported herein versus the commercially available contour PVA (PSD: 355–500 µm). Both particles exhibit high irregularity in shape, with contour PVA possessing high levels of porosity by comparison. In general, the experimental embolic particles were observed to exhibit a smoother surface topography, coupled with a narrower particle size distribution (*possibly due to the non-aggregating nature of the materials composition compared to PVA*).

### 2.3. Material Response

#### 2.3.1. *In Vitro* Dissolution

**Figure 2:**
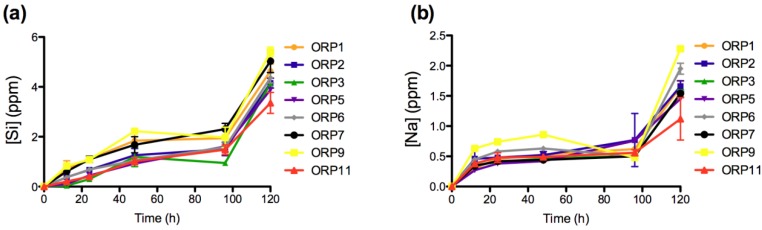
Ion release levels for (**a**) Si; and (**b**) Na release of the eight embolic particles with respect of time at 12, 24, 48, 96 and 120 h.

**Figure 3:**
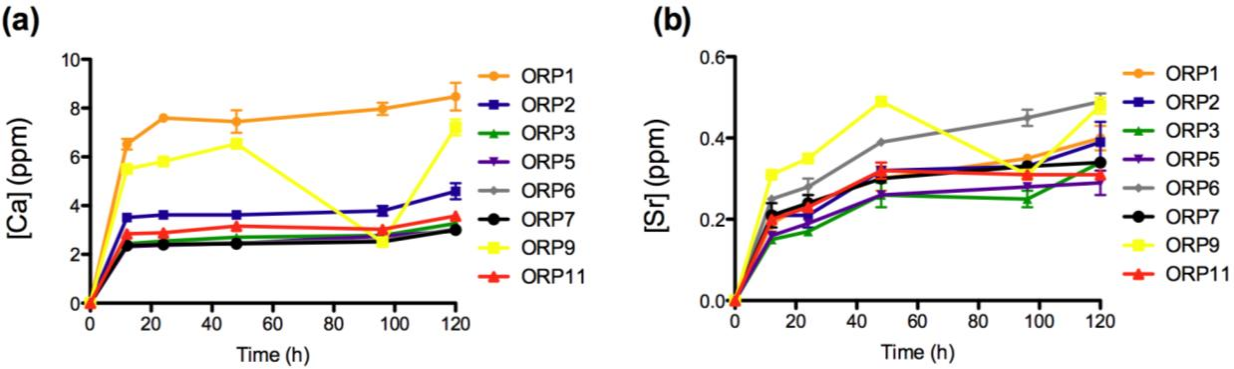
Ion release levels for (**a**) Ca; and (**b**) Sr release of the eight embolic particles with respect of time at 12, 24, 48, 96 and 120 h.

**Figure 4:**
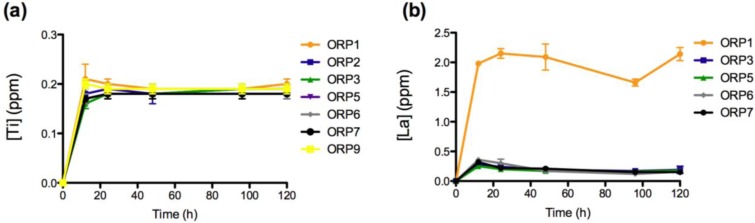
Ion release levels for (**a**) Ti; and (**b**) La release of the eight embolic particles with respect of time at 12, 24, 48, 96 and 120 h.

**Figure 5:**
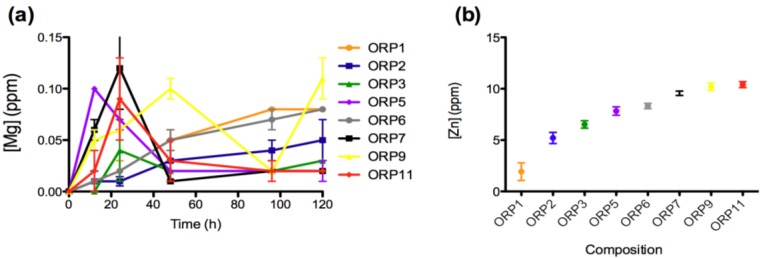
Ion release levels (12, 24, 48, 96 and 120 h) for (**a**) Mg; and (**b**) mean (±SD) release levels for Zn of the eight embolic particles.

The materials response (generation of dissolution by-products) was evaluated after short-term exposure (12, 24, 48, 96 and 120 h) to a simulated physiological environment. Time-dependent ion release levels provide an insight to the materials *in vitro* dissolution behaviors with respect its Si^4+ ^(0.04–5.41 ppm), Na^+^ (0.27–2.28 ppm), Ca^2+^ (2.32–8.47 ppm), Sr^2+^ (0.15–0.49 ppm), Ti^4+^ (0.16–0.20 ppm), La^3+ ^(0.025–0.214 ppm), Mg^2+^ (0.01–0.11 ppm) and Zn^2+^ (1.93–10.40 ppm) components. All ions with the exception of Zn^2+ ^demonstrate immediate release as soon as the specimens were immersed in solution. Ion release behaviors for Ca^2+^, Sr^2+^, Ti^4+^, La^3+^ and Mg^2+^ are deemed representative of the heterogeneous glass dissolution model [28] (see Figure 2, Figure 3, Figure 4, Figure 5). Figure 5b compares the effect of each materials composition on Zn^2+^ release levels at 120 h. Concentrations at t = 0 h correspond to the assumed values of the solutions prior to immersion of the 8 ORP compositions (subsequent to incubation). 

The rate of dissolution of the network forming silica species from the 8 material compositions increases significantly upon immersion into solution up to 12 h (*p < 0.005 for ORP2 and p < 0.0005 for ORP1, 6, 7 and 9*), with the exception of ORP3, 5 and 11 (which increase significantly after 24 (*p < 0.0005 for ORP5*) and 48 h (*p < 0.0005 for ORP 3 and 11*). Si^4+^ release is later observed to significantly increase (*p < 0.0005)* simultaneously for all compositions between the final time points (96 to 120 h) assessed. The Si^4+^ release levels observed herein are unlikely to have adverse effects from a systemic perspective, since Si^4+^ is frequently absorbed from the diet as orthosilicic acid at levels beyond this threshold [29]. Of importance, silicon has found widespread use as an embolic material in the form of a cylindrical plug for the cerebral arterial occlusion of a canine model [30,31], as Y-shaped particles for partial splenic embolization, slowly occluding splenic arterial branches to produce ischemia in a gradual fashion and minimize the pain after embolization [32] and most recently as porous micro and nanospheres (multi-stage delivery vehicle) for treatment of various cancers [33,34].

The rate of dissolution of the network modifying Na_2_O component from the 8 material compositions is also found to increase significantly upon immersion into solution up to 12 h (*p < 0.05*). In addition, significant increases (*p < 0.05*) in Na^+^ release were also observed between 12 and 24 h for all compositions (with the exceptions of ORP2 and 11). No further significant differences were detected between all compositions until 96 to 120 h. Na_2_O content in silicate glasses has been previously reported to impart a positive effect on cell viability by facilitating an enhanced specific surface area [29]. Additionally, it can be anticipated that the Na^+^ release levels observed are unlikely to lead to any systemic complications, since the release levels reported herein fall well below human plasma concentrations of approximately 3200 ppm (142 mM) [35]. Recently, micro-algae sodium microspheres (marketed as KMG microspheres) have been investigated in pre-clinical trials for their potential arterial occlusive activity as embolic agent in UFE: focusing on their impact on ovarion function and subsequent pregnancy [36]. Sodium tetradecyl sulfate compound however is a commonly used form of sclerosant liquid agent for treatment (*permanent occlusion*) of arteriovenous malformations in small vessels [37]. Interestingly, composition ORP5 represented the only composition to show a significant increase in both Si^4+^ and Na^+^ release between all dissolution time points, indicating a high level of controlled release for these ions based on this material formulation.

The rate of dissolution of the network modifying CaO component from the 8 material compositions also increases significantly upon immersion into solution up to 12 h (*p < 0.0005*) and later from 96 to 120 h (*p <* 0.0005 (*with the exception of ORP1*)). No significant differences were observed for ORP2, 3, 7 and 11 from 12 to 24 h and for ORP1, 2 and 7 from 24 to 48 h. The remaining compositions exhibited significant increases in Ca^2+^ release between these time-periods, with the exception of ORP9 that exhibited a notably significant decrease (*p <* 0.0005). Similar to Si^4+^ and Na^+^ release, ORP5 represents the only composition to show a significant increase between all time-periods to indicate a high level of controlled Ca^2+^ release for this material formulation. This is likely due to the formation of a hydrogel like layer on the full surface area of the glass particle, as a result of initial Ca^2+^ release (*from the particle*) in exchange for H_3_O^+^ in the incubation media, to precipitate onto the entire surface of the particle; and further impact upon the rate of ion release from the particle [28,38,39]. This gel layer formation is dependent on the glass composition; with different compositions having the capacity to form various multi-layered structures. Further dissolution is found to result in the mass transport of alkali ions from the center of the particle glass through the hydrogel rich layer into the aqueous media, causing a reduction in the rate at which dissolution of the particle occurs. As immersion time increases, the release of the glass constituents depends on the penetration of water molecules into the subsurface layer, thus the alkali ions may be mobilized again with continued dissolution of the glass. In addition, these results also correlate well against Q^n^ species defined previously by the authors for this glass series [19]. The presence or absence of calcium determines the activation, activity, oligomerization, and stability of blood coagulation factor XIII (fXIII) [40], with the normal concentration for total serum Ca^2+^ reported to vary between 8.5 to 10.5 mg/dL [41]. Physiologically, Ca^2+^ is required for fXIII activation and for transglutaminase activity [40]. In the blood, activation of circulating fXIII requires thrombin cleavage, calcium ions (1.5 mm) [42] and fibrin(ogen). High levels of calcium (>50 mm) can activate factor XIII without the use of thrombin [40], and it has recently been shown that platelet fXIII can be activated nonproteolytically *in vivo* [43]. As a result, the low levels of Ca^2+ ^reported herein are unlikely to lead to any systemic complications, but nevertheless may favor the likelihood of a thrombus formation.

The rate of dissolution of the network modifying SrO component from the 8 material compositions increases significantly upon immersion into solution up to 12 h (*p <* 0.0005), from 12 to 24 h (*p < 0.05; with the exception of ORP2 and 3*) and later from 24 to 48 h (*p <* 0.005). From 48 to 96 h, ORP1, 6 and 7 exhibit significant increases (*p < 0.05*) in concentrations of Sr^2+ ^release while ORP9 displays a notably significant decrease (*p < 0.0005*). Finally, from 96 to 120 h, significant increases (*p < 0.05*) in Sr^2+^ release were observed for ORP1, 2, 3, 6 and 9. Similar to Ca^2+ ^release, an initial burst of Sr^2+ ^released from all compositions (*excluding ORP6 and 9*) was evident up to 48 h, ending gradually to maintain peak ion release levels for the remainder of the study. Interestingly, in terms of the overall structure of ion-bound blood coagulation fXII, calcium and strontium are found to bind in the same location [40]. However, while the same molar concentrations for SrO and CaO_2_ are present for all nominal experimental embolic compositions, Ca^2+^ is seen to release at a factor of up to 10 times as much Sr^2+^; a factor that may compensate for the low levels of Ca^2+^ eluted from the compositions to initiate activation of fXII.The rate of dissolution of the network forming TiO_2_ species from the 8 material compositions increased significantly upon immersion into solution up to 12 h (*p < 0.0005*). No significant differences were observed however for any of the compositions between all subsequent time-points evaluated thereafter (*except for ORP3 significantly increasing* (*p < 0.05*) *from 12 to 24 h*); a feature likely attributable to the hydrogel like layer encapsulating the entire particle surface area. Normal serum Ti levels are <150 mg/L [44], with the observed concentrations reported in this study falling far below this level to lead to systemic complications. TiO_2_ has been shown to be highly thrombogenic, a feature associated with superior osseointegtration in hard tissue implants [45,46]. As such, Ti^4+^ dissolution products emanating from embolic particles such as those developed herein may be perceived to offer occlusive capability in the field of peripheral vascular embolization processes. Their effect however has yet to be published in the literature, but may warrant such an investigation.

The ionic dissolution results show a notably significant increase (*p < 0.0005*) in the concentration of the network modifying La_2_O_3_ component for all compositions upon immersion in solution up to 12 h, with ORP5, 6 and 7 showing subsequent significant decreases (*p < 0.05*) from 12 to 24 h. No significant differences were observed between the remaining time-points evaluated (*with the exceptions of ORP6 significantly decreasing* (*p < 0.0005*) *from 24 to 48 h; ORP1 and 7 significantly decreasing* (*p < 0.005*) *from 48 to 96 h and ORP1 subsequently increasing* (*p < 0.0005*) *from 96 to 120 h*). ORP1 and 6 initially contain 0.137 and 0.188 mole fraction La^3+^, respectively, while the remaining compositions reported, each comprise of 0.068 mole fraction. Furthermore, the only difference occurring between compositions ORP5 and ORP6 is a direct substitution of Zn^2+^ for La^3+^, which is shown to result in a slow down in the release of La^3+^. The higher release levels for ORP6 may be easily attributed to its crystalline structure, where La_2_O_3 _is seen to heavily act as a network modifier in this zinc-silicate based system [47]. Previously, several lanthanide ions have been shown to replace calcium ions during fXIII activation (10–40 μM), with much of these effects occurring with Ca^2+^ present in the millimolar range, and with the lanthanide ion in the micromolar range [40]. Furthermore, La_2_O_3_, as a radioactive rare earth element (REE), has recently been used in the diagnosis and treatment of cancer, to investigate its effects on tumor development and growth. Substantial evidence exists showing that REEs inhibit proliferation and induce apoptosis in certain cancer cell lines [48]. Su *et al*. [48] report the effect of lanthanum citrate (LaCit), in which lanthanum exists as ionic state (La^3+^), for which it was found to induce anoikis (programmed cell death) in Hela cells at a dose between 0.001 and 0.1 mmol/L. As such, the inclusion of La_2_O_3_ in the experimental embolic particles indicates that it may offer future therapeutic effects for the potential treatment of HCC. 

The rate of dissolution of the network modifying Mg^2+^ component from the 8 material compositions is slow compared with the dissolution of other ions. The slow release of Mg^2+ ^into the solution suggests that it is strongly chelated by the silicate network [49]. In summary, ORP5, 7 and 9 were the only compositions to demonstrate significant increases in the concentration of Mg^2+ ^from immersion into solution up to 12 h. Subsequently, ORP3, 5, 7 and 11 is observed to exhibit significant increases (*p < 0.005*) from 12 to 24 h. Furthermore, a significant decrease in Mg^2+ ^is detected from 24 to 48 h (for ORP5, 7 and 9 (*p < 0.0005*) and ORP11 (*p < 0.005*)) and later from 48 to 96 h (for ORP9 (*p < 0.005*)). ORP1 and 6 (*glass-ceramic compositions*) exhibit significant increases (*p < 0.005*) between the same time-points. No significant differences are noted from 96 to 120 h with the exception of ORP9, which continued to significantly decrease (*p < 0.0005*). In summary, wIth the dissolution by-products for Mg^2+^ not exceeding an average of about 0.1 ppm, the reductions observed between 24 to 48 h or 48 to 96 h may be considered negligible. The normal concentration of serum Mg is in the range of 1.5–2.3 mg/dL [50], with a Mg concentration >3 mmol/L reported as having a statistically significant but probably clinically unimportant effect on coagulation with Mg unlikely to have adverse effects in patients in whom Mg is being used therapeutically. Mg has found use however as a thrombogenic material that favors intrasaccular [51] clotting in aortic aneurysms, with further investigations in haemangiomas and lymphangioma tumors, based on the obliteration of the tumor by enhanced blood clotting from the mechanical destruction of the endothelium and the septa of the tumor [52]. Furthermore, Mg salts both topically and parenterally have been reported to suppress thrombus formation; although they have also been linked to increased concentration of ADP, which is required to initiate thrombus production at minor injury sites [53]. While excessive levels of MgO in the blood can result in serious side effect such as dangerously low blood pressure and irregular heartbeat [54], the inclusion of MgO in the experimental embolic particles may offer benefits in terms of its efficacy, whilst not evoking such adverse reactions on account of its low level of dissolution from the glass system. 

Zn^2+^ release was not evident in this study until 120 h immersion in solution. A compositional trend for Zn^2+^ release however, was observed in the following order (from highest to lowest release levels): ORP11 > ORP9 > ORP7 > ORP6 > ORP5 > ORP3 > ORP2 > ORP1 (Figure 4b). Indeed compositions ORP9 and 11 contain the highest starting Zn^2+^ content (*both at 0.290 mole fraction*), while ORP1 contains the second lowest Zn^2+^ content at 0.137 mole fraction. ORP6 (*containing the lowest Zn^2+^ content at 0.068 mole fraction*) however, is found to release higher levels of Zn^2+ ^over the experimental embolic particles containing lower quantities of Zn^2+^ (0.188 to 0.240 mole fraction). This may be attributable to its predominantly glass-ceramic structure containing crystalline phases that act to increase its dissolution rate [55]. Human Zn blood plasma levels have been shown to be approximately 6.4 ppm [56], with Zn^2+^ release from biomaterials in the range 3–7 ppm have shown antibacterial efficacy [57]. Hence, it may generally be accepted that any glasses releasing Zn^2+^ levels within these ranges may offer similar beneficial effects. Thus, zinc-containing glasses, ORP2, 3 and 5 may have potential therapeutic efficacy in this regard. Limited literature however still exists on the release of Zn^2+^ from biomaterials *in vivo*, in particular those focused on bioactive glasses. So further investigation into whether high Zn^2+^ levels cause *in vivo* cytotoxicity is warranted to verify the efficacy of the high zinc-containing glasses remaining in this series (such as, ORP7, 9 and 11). Interestingly, Aina *et al*. [58], have shown that increased Zn^2+^ content in Zn-doped silicate based glasses may reduce the overall leaching activity of the glass on the basis of molecular dynamics modeling. The first step of bioactive glass dissolution, assumed to involve the rapid exchange of Na^+^ for H_3_O^+^ ions present in the solution, is hindered by the progressive obstruction of the percolation channels used for Na^+^ ion diffusion in Zn-containing glasses (*with this obstruction activity a function of the Zn concentration*). The antibacterial effects of Zn^2+^ have been evaluated by broth dilution methods, in which bacterial growth was inhibited in the most concentrated Zn^2+^ oxide (0.719 ppm and 1.046 ppm) suspension under the same conditions [28]. For these applications, the appropriate Zn^2+^ concentration was found to range between 0.065 ppm and 19.614 ppm. Unfortunately, there is limited information concerning the effects of Zn^2+^ release in transarterial embolization indications. 

In summary and based on ion release levels from the purely amorphous compositions alone (*i.e.*, excluding ORP1 and 6); composition ORP9 (containing no La_2_O_3_) released the greatest amounts of Si^4+^, Na^2+^, Ca^2+^, Sr^2+^, Ti^4+^, Mg^2+^ and Zn^2+^ at the final time-point of the study. By comparison, composition ORP5 provides the slowest dissolution properties in terms of Ca^2+^, Sr^2+^, Ti^4+^, La^3+^ and Mg^2+^ at 2.96, 0.29, 0.18, 0.16 and 0.02ppm respectively. It also releases the lowest levels of Si^2+^ and Na^2+^ (3.88 and 1.45 ppm, respectively) at the final time-point of the study after composition ORP11 (3.36 and 1.12 ppm, respectively). Finally, as exhaustive ion release levels have yet to be realized, these materials warrant further investigation over longer time-periods (up to 60 days) in order to understand fully their long-term dissolution kinetics.

#### 2.3.2. Cell Viabilities (MTT Assay) of the Embolic Particles

**Figure 6:**
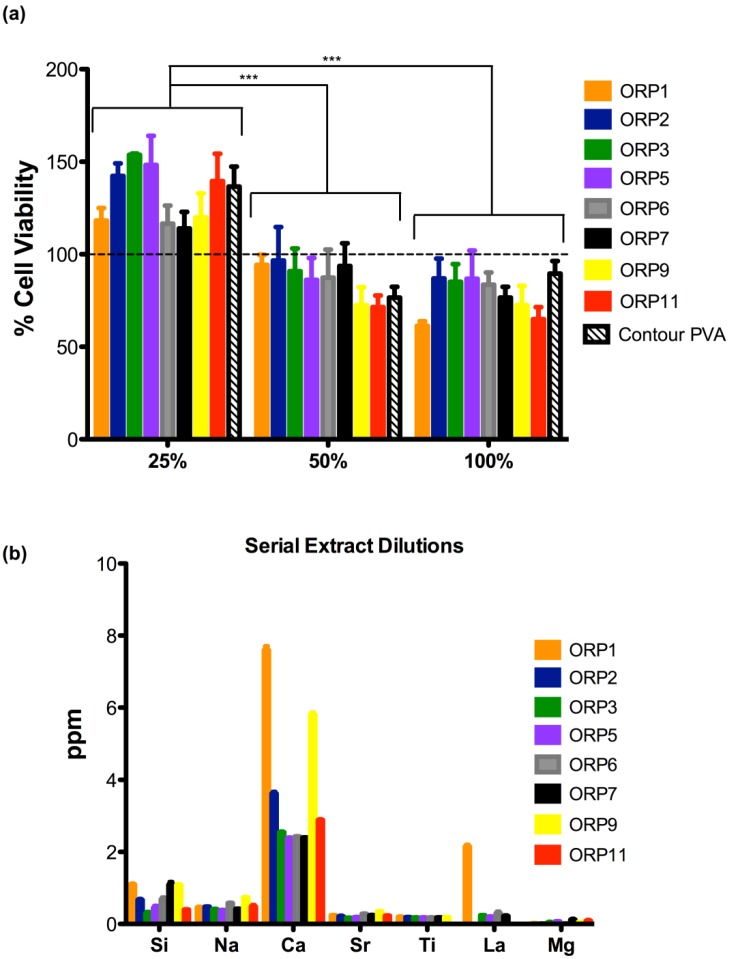
(**a**) Cell viability in the presence of ORP1-3, ORP5-7, ORP9 and ORP11 and contour PVA for 24 h embolic particle extracts at 25, 50 and 100% serial dilutions. Results represent mean ± SD (no significant statistical differences (*ρ* > 0.05) were observed between any of the extracts tested). Where *** represents *ρ* < 0.0005; and (**b**) Dissolution by-products present in each extract in correlation to the cell viabilities reported in (**a**).

The second part of this study evaluated the cytotoxicity of serial extract dilutions to substantiate the identification of a preferable composition for pre-clinical use. The viability of cells exposed to extracts derived from each experimental embolic particle at: 25% (*113.9* ± *15.49 to 153.7* ± *1.25%*); 50% (*71.44* ± *10.90 to 96.60* ± *31.44%*); and 100% (*61.31* ± *4.33 to 86.72* ± *26.47%*) serial extract dilutions after exposure to 24 h extracts versus contour PVA are presented in Figure 6a. All experimental embolic particles demonstrated mean cell viabilities higher than or equivalent to contour PVA. However, no significant differences were observed amongst all experimental embolic particles including contour PVA at any of the three serial dilutions performed. As expected, mean cell viabilities between the serial dilutions, showed statistically significant differences (as identified in Figure 6a) between 25 and 50% as well as 25 and 100%. According to ISO 10993 [1], 50% extract dilution’s should have at least the same or a higher viability than the 100% extracts; otherwise the test should be repeated. Our observations confirm that the cell viabilities between 50 and 100% extract dilution’s remain at least the same or higher percentage cell viability. At 25% extract dilutions however, all experimental ORP extracts showed enhanced cell viabilities exceeding 100% versus extract dilutions at 50 and 100%. 

Higher extract dilutions exhibiting decreased cell viabilities may be attributed to the dissolution of glass in an aqueous media, leading to high pH values in its surrounding environment. Reducing the concentration of the extract dilutions from 100 to 25% may minimize the rate at which the pH is affected to support the growth of NIH-3T3 cells, indicative of the data presented in this study demonstrating induced proliferation and growth of cells at 25% extract dilutions (exceeding 100% cell viability). Of note is the presumption that enhanced cell viability evident by the induced cell proliferation corresponds to a beneficial result. Genotoxic effects can cause cell DNA damage, resulting in malfunctions within the regulation of the cell cycle and subsequent uncontrolled proliferation of mutated cells [59]. Classic cytotoxicity tests, such as that used in this study, do not differentiate between normal and aberrant cell growth. In order to fully assess the materials examined here and to support the findings that the levels of ions released enhance biocompatibility, relevant genotoxicity testing is necessary. Figure 6b indicates the corresponding dissolution by-products present for each of the compositional extracts that were tested *via* MTT assay, presented in Figure 6a. 

Previous studies by the authors have indicated that specific combinations and concentrations of ions enhance cell proliferation of various cell types in comparison to exposure of individual ions of equal concentration [59]. Thus, it may be feasible to suggest that the enhanced cell viabilities of the experimental embolic compositions exposed to the NIH-3T3 fibroblast cells in this study are due to a synergistic effect between certain concentrations of ions [60]. In a previous study, the authors demonstrated *via* a mixture design approach the top five most influencing combinations of ions (including any ionic interactions) affecting cell viabilities for this zinc-silicate glass system as follows (from highest to lowest): ZnO*TiO_2_ > TiO_2_ > ZnO*SiO_2_ > SiO_2_ > ZnO*La_2_O_3_. However, the specific mechanisms of the specific stimulatory effect of ion combinations remain unknown. When related to the ion release profiles, extract ORP1 contains significantly higher concentrations (*p < 0.005*) of Si^4+^ (1.10 ± 0.04 ppm) and Ca^2+^ (7.60 ± 0.18 ppm) in comparison to all remaining experimental extracts (*excluding ORP7 and 9, where no significant differences were observed with regard its concentration of Si*^4+^). Significantly higher concentrations (*p < 0.0005*) of La^3+^ (2.15 ± 0.08 ppm) were also observed in extracts of ORP1 compared to all remaining extracts whose compositions also eluted La^3+^ (ORP3, 5, 6 and 7). Similarly, extracts of ORP9 were found to contain significantly higher concentrations (*p < 0.005*) of Si^4+ ^(1.08 ± 0.04 ppm), Na^+^ (0.74 ± 0.01 ppm), Ca^2+^ (5.81 ± 0.01 ppm) and Sr^2+^ (0.35 ± 0.01 ppm) combined with significantly lower concentrations (*p < 0.005*) of Mg^2+^ (0.06 ± 0.00 ppm). By contrast, extracts of ORP3 and 5 were observed to contain the least amount of Si^4+^ (at a mean average of 0.1510–0.2264 ppm) and Ca^2+^ (at a mean average of 2.38–2.55 ppm) yet possessed the highest levels of mean cell viabilities (85.10 to 153.69%) compared to ORP1, 9 and 11 (61.31 to 118.20%) at 25 and 100% serial dilutions; attributable to the high levels of tissue culture water present in their MTT extracts.

### 2.4. Host Response

#### 2.4.1. Fg Conformational Changes in Dissolution Extracts of the Embolic Particles

**Figure 7:**
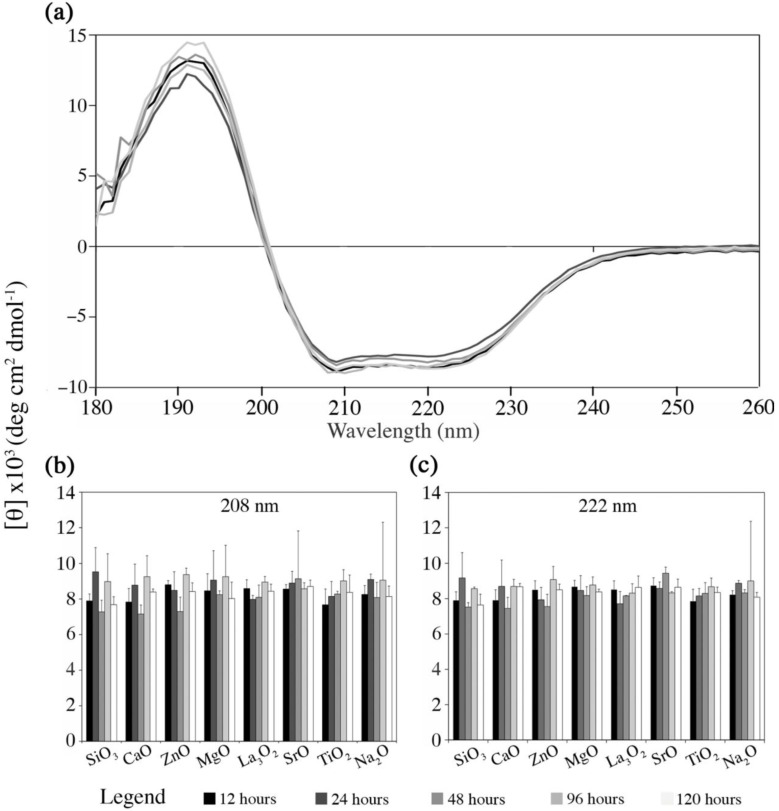
Changes in Fg conformation monitored by far-ultraviolet circular dichroism (CD) spectroscopy as a function of the ionic dissolution products with respect to time. (**A**) Example of CD spectra for ORP5 over time; (**B,C**) Intensity (mean of 3 replicates, blank subtracted; average deviation shown) at the minima at ~208 and 222 nm of Fg CD spectra for each ionic dissolution product. All data is reported in mean residue ellipticity [θ]. The provided legend applies for all three panels.

The final part of this study observed the likely host response, by examining the effect of the materials dissolution by-products on Fg conformation, and the preferred material (ORP5) particles surface on haemocompatbility. Figure 7 presents the changes in Fg conformation, as monitored by far-ultraviolet CD spectroscopy as a function of the solutions of each embolic extracts with respect to time (analyzed in detail in the previous section (Section 2.3) in terms of their dissolution rates over time). Figure 7a illustrates the CD spectra collected from the preferred experimental embolic particle ORP5 as a function of time and is representative of the CD spectra obtained from ORP1, 2, 3, 6, 7, 9 and 11. Figure 7b,c compare the intensities at the minima (~208 and 222 nm) of Fg CD spectra for each ORP compositions ionic dissolution products. Quantitatively, there was no significant difference identified in terms of the Fg state between experimental embolic particles.

An evaluation of the potential effect of dissolution by-products on the secondary structure of Fg (*an essential precursor protein for clot formation*) was undertaken. As stated previously, unwanted changes in Fg conformation may adversely result in site-specific thrombus formation [22] onto the surface of the dissolution by-products as opposed to the surface of the implanted particle itself, as required to ultimately limit blood supply to the intended target area. Fg is a 340 kDa water-soluble protein; consisting of two units with three pairs of non-identical polypeptide chains, Aα-, Bβ- and γ-chains held together by S-S disulfide bridges [61]. It is theoretically accepted that this peptide chain undergoes spontaneous folding in aqueous solutions, to adopt a native conformational form corresponding to an energy minimum. In our investigations, Fg was dissolved in a buffer solution because of its native tertiary structure. Presumably, if glass dissolution by-products are incorporated into such a medium, then the energy medium of the protein should be affected as a result of non-specific interactions of the macromolecules on the surface of the dissolution by-products. Previous studies have indicated that protein-silicone complexes may be explained by the silicone’s high affinity for hydrophyllic compounds through the interaction with the siloxane groups –Si–O–Si– [61]. Changes in the structure of Fg distal to the embolic site could result in major secondary effects by inducing imbalances in hemostasis. No significant changes to Fg structure however were observed by CD (Figure 7) in our evaluation. We therefore conclude that the novel embolic particles presented herein offer promise as biocompatible materials, in this regard. 

As with any study involving PBS and Fg, intrinsic limitations exist that must be acknowledged, such that the impact of the discussed data is as relevant as possible, including (but not limited to):

(i) Mimicking the protein environment using PBS solution inherently draws limitations, since its use as a medium has an insufficient buffer capacity in terms of the medium pH and Ca^2+^ concentration, for which these factors in the real physiological environment are kept homeostatic *via* the circulatory system. PBS instead of simulated body fluid (SBF) was used in order to avoid the interference with UV signals, which is derived from the organic contents in SBF (Tris);
(ii) In addition, protein complexation within the physiological environment was extensively simplified by using diluted mono-protein solution (Fg). This is due to the difficulties involved in separating and quantifying mixed protein solution.

#### 2.4.2. Platelet Lactate Dehydrogenase (LDH) of the Embolic Particles

**Figure 8:**
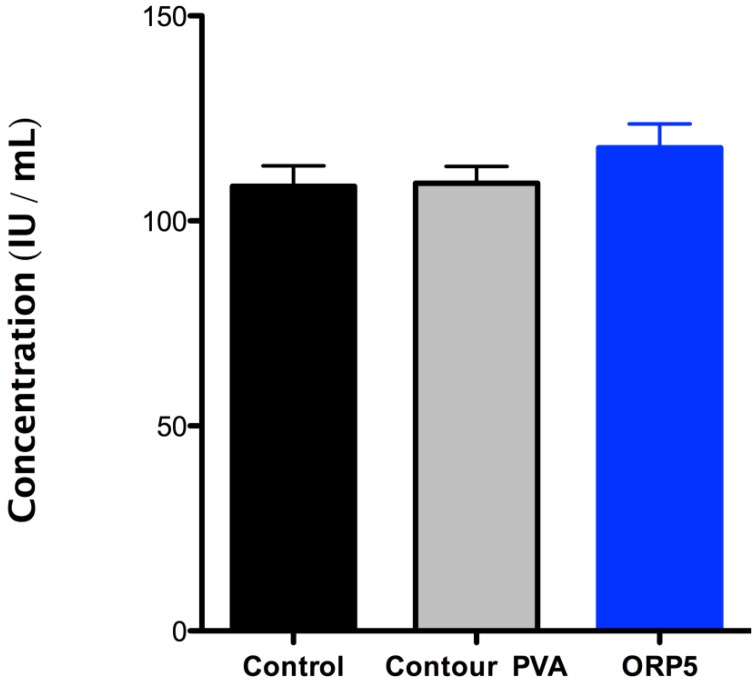
The adhesion of human platelets in its pre-incubated state (control) and on contour PVA and ORP5, determined by LDH assay. The levels of platelet adhesion (in mean values) was higher for ORP5 than for contour PVA, with no significant difference between all three groups tested.

The adhesion of platelets onto the surface of the ORP5 particle was confirmed with LDH assay (see Figure 8), which revealed higher platelet adhesion onto ORP5 than either contour PVA or PRP pre-incubation (control). Of note, according to ISO10993-Part 4, variables that shall be considered when using in vitro test methods for compatibility using blood, include haematocrit, anticoagulants, sample collection, sample age, sample storage, aeration and pH, temperature, sequence of test versus control studies, surface-to-volume ratio, and fluid dynamic conditions (especially wall shear rate). Tests are also required to be performed with minimal delay, (within 4 h), since some properties of blood change rapidly following collection. Using different blood from patients therefore provides for a more robust approach in terms of allowing extraneous variables to be considered in the final study design, whilst also serving to improve reliability in the final data obtained (considering no significant differences were observed between all three groups tested). 

The experimental embolic material ORP5 has previously been established as the preferred composition (*in terms of its structure and cytocompatibility* [19]) through experimental design methodologies, warranting further investigation. The *in vitro* dissolution studies discussed herein confirm its suitability to provide long-term occlusion to targeted blood vessels. Furthermore, evaluating the materials host response *in vitro* for platelet activity by LDH assay, confirmed a higher adsorption of human platelets (*as a mean per sample tested only*) onto ORP5 than contour PVA. Quantitatively however, there was no significant difference noted between platelet adhesion between both groups compared to PRP in its pre-incubated state. Surface topographies are known to be a major influencing factor in affecting platelet adhesion levels, with rough surfaces exhibiting an increase in fibrinogen adsorption and platelet adhesion at early blood contact times [62]. While the preferred adhesion of platelets onto ORP5 may not be fully attributed to its topographical difference from contour PVA (*ORP5 having a smoother/angular morphology to contour PVA, as demonstrated in* Figure 1), it may be due to intrinsic characteristics of the material composition. The presence of Ca, for example is documented to potentially trigger the adhesion of platelets to secrete certain growth factors [62], with Si, Ti and Na also each reported to play important roles in triggering platelet adhesion [51]. Inherently, all the experimental embolic compositions reported in this study contain such metal oxides as part of its main compositional variants. As such, the controlled release of metal ions (*via* mechanisms such as: hydration, hydrolysis of the ionic–covalent network, pore-opening and exchange between alkali or alkaline-earth ions and protons in solution) from ORP5 may induce adhesion of platelets to allow platelets to contract and release the contents of their granules into the extracellular environment [51]. These granular extracts may then activate other platelets, provoke irreversible platelet aggregation and lead to the formation of a platelet thrombus [22], an essential property for full occlusion of blood vessels. However, as with any study involving blood studies, intrinsic limitations exist which must be acknowledged such that the impact of the discussed data is as relevant as possible, including (but not limited to):

(i) It should be noted that the mechanisms of degradation may not be the same for all materials at low pH as they are at blood pH (approximately pH 7.35 to pH 7.45).
(ii) While it is recognized that additional biological factors such as enzymes and proteins can alter the rate of degradation, degradation by such outside factors is not addressed in this Part of ISO 10993.

Nonetheless, as an extreme condition for the production of possible dissolution by-products has been used in our experimental methodology, this “worst case scenario” may serve as a screen for these materials.

## 3. Experimental Section

### 3.1. Materials Synthesis

Eight embolic particle compositions (Table 1) of irregular morphology were prepared, based on a design of mixtures (DOM) experimental design (using Design-Expert 8.0.4 software, Stat-Ease Inc., Minneapolis, MN, USA) as described elsewhere [19]. The designations for each embolic composition (ORP1-3, ORP5-7, ORP9, ORP11) are based on original designations from the full DOM design space and are maintained in this study for ease of reference with the previous literature. Briefly, analytical grade reagents: silicon dioxide, calcium carbonate, zinc oxide, magnesium oxide, lanthanum (III) oxide, strontium carbonate, titanium dioxide and sodium carbonate (Sigma Aldrich, Canada) were weighed out and mixed to a homogeneous blend in a plastic container (NalgeneR, Sigma Aldrich, Canada) for 1hr. Each batch of powder was placed in platinum crucibles (50 mL), then fired (1480 °C, 1 h) using a Bench-Top High Temperature Muffle Furnace (EQ-KSL, MTI Corporation, Richmond, CA, USA) and shock quenched into distilled water. The resulting glass frit was dried in an oven (120 °C, 1 day), pulverized in an agate planetary mill (Pulverisette 7; Laval Labs Inc., Laval, QC, Canada) and sieved to retrieve irregular shaped particle in the range 45–212 µm and 300–500 µm (equivalent to a clinically useful particle size distribution (PSD) for clinically used embolic particles). All experimental ORP powders were subsequently stored in dry desiccators for subsequent evaluation. 

#### Commercial Control

Irregular shaped contour PVA embolization particles (Boston Scientific, Cork, Ireland) of 45–150 µm and 355–500 µm particle size distributions were used as commercial controls. *In vitro* analysis of contour PVA *versus* the experimental ORP particles was undertaken for cytotoxic (Product Lot No’s. 13473927 and 13599201) and haemocompatibility (Product Lot No. 14575090) evaluations. 

### 3.2. Material Morphology and Particle Size Distribution

For a comparison of the particle morphologies, contour PVA (355–500 µm; Product Lot No. 14206690) and experimental ORP particle (355–500 µm) were mounted onto 10 mm diameter × 3 mm high Al stubs and coated with ~27 nm platinum using a gold-sputter coater (SC7640, Fisons Instruments, Ipswich, UK). The morphology of the particle was subsequently observed by field emission scanning electron microscopy (Hitachi S-4700 FEG-SEM) at an accelerating voltage of 5.0 kV under secondary electron mode. The particle size distributions were performed for the same particles with a Malvern Mastersizer 3000, which uses a laser diffraction technique with multiple light detectors to obtain the diameters of particles. Due to a limitation associated with laser diffraction technique (*i.e., optimizing the intensity*), the measurements were normally run with 5 diluted sub-aliquots of particle suspensions, to minimize errors.

### 3.3. Material Response Evaluation

#### 3.3.1. Preparation of Embolic Extracts

To examine the dissolution behavior of each embolic particle; a short-term dissolution study was set-up to examine the material response in a simulated physiological environment. 0.1g of each embolic material (ORP1-3, ORP5-7, ORP9, ORP11 as prepared in Section 3.1) was immersed in 10 mL (1% w/v) of sterile tissue culture water (Sigma-Aldrich, Canada) (n = 3) for 12, 24, 48, 96, and 120 h time periods. Each specimen was stored in polypropylene tubes maintained at 37 °C in a shaking waterbath (Stuart Sb40, Techne Inc, Burlington Township, NJ, USA), and agitated at 2 Hz (longitudinal movement) [21]. After each storage period, individual extracts derived from the embolic particles were filtered using a sterile 0.20 μm filter (Sarstedt, Saint-Léonard, QC, Canada). Following that, 3 mL of each filtrate was diluted to 30 mL extract with tissue culture water and stored at 4 °C for subsequent ionic content analysis. 

#### 3.3.2. Ionic Content Analysis

The Si^4+^, Na^+^, Ca^2+^, Zn^2+^, Ti^4+^, La^3+^, Sr^2+^, and Mg^2+ ^concentrations for each extract was analyzed using inductively coupled plasma atomic emission spectroscopy (ICP-AES, PerkinElmer Optima 3000, PerkinElmer Inc., Wellesley, MA, USA). The absorption wavelengths used for the determination of each element are reported in Table 2. Before each cycle of measurement, calibration curves were obtained by preparing standard solutions containing Ti^4+^, La^3+^, Sr^2+^, and Mg^2+ ^and a separate set of standard solutions containing Si^4+^, Na^+^, Ca^2+^ and Zn^2+^ (as obtained from JVA Analytical Ltd, Dublin, Ireland) at concentrations reported in Table 3, respectively. Standard sample concentrations were measured periodically to ensure the accuracy of the calibration curve. ICP-AES analyses for each extract (prepared as per Section 3.3.1) were performed in triplicate (n = 3 (extracts per condition), with 3 ICP-AES analyses performed on each extract).

**Table 2:** Emission lines used for the ICP measurements.

Element	Absorption wavelength	Lower limit	Upper limit	Background correction
Si^4+^	288.158	288.073	288.256	±0.026
Na^+^	330.237	330.136	330.348	±0.030
Ca^2+^	396.847	396.679	397.039	±0.072
Zn^2+^	334.501	334.400	334.614	±0.031
Ti^4+^	337.279	335.188	334.810	±0.031
La^3+^	407.735	407.971	407.596	±0.075
Mg^2+^	279.553	279.646	279.399	±0.026
Sr^2+^	421.552	421.759	421.371	±0.078

**Table 3:** Standard concentrations used for the ICP measurements.

**Standard**	**Chemical element**			
	Si^4+ ^(mg/L)	Na^+ ^(mg/L)	Ca^2+ ^(mg/L)	Zn^2+^ (mg/L)
1	2	1	0.5	1
2	4	2	1	2
3	10	4	3	4
**Standard**	Ti^4+^ (mg/L)	La^3+^ (mg/L)	Mg^2+^ (mg/L)	Sr^2+^ (mg/L)
4	0.1	0.1	0.1	0.1
5	1	1	1	1
6	10	10	10	10

#### 3.3.3. *In Vitro* Biological Evaluation of Materials

*In vitro* cytocompatibility as it pertains to each material was evaluated using the MTT (3-(4,5-dimethylthiazol-2-yl)2,5-diphenyl tetrazolium bromide) assay to measure cell survival/proliferation, and ultimately verify low levels of cytotoxicity between the experimental compositions versus the commercial predicate, contour PVA. Briefly, experimental embolic particles were sterilized by autoclaving (AMSCO, Medallist) at 121 °C/25 bar for a period of 20 min. prior to incubation. Specimen extracts for each material were prepared as per the dissolution study protocol (see Section 3.3.2). After a 24 h incubation period (at 37 °C), specimens were subsequently filtered using a sterile 0.20 μm filter (Sarstedt, Canada), with filtrates stored at 7 °C prior to *in vitro* evaluation. The commercial material was received sterile and handled under aseptic conditions for this analysis.

#### 3.3.4. Fibroblast Cell Culture

Immortalized rat fibroblasts (NIH-3T3; American Type Tissue Collection, Manassas, VA, USA) at passages 15–20 were used for experiments. The cells were grown in 75 cm^2^ tissue culture flasks in Dulbecco’s Modified Eagle’s Medium (DMEM) supplemented with 5% fetal calf serum (FCS; heat-inactivated at 56 °C for 60 min). Cells were passaged twice weekly at 70% confluence, using 1 mL of 0.25% trypsin-EDTA to detach cells, and resuspended in 19 mL of DMEM. Flasks were maintained in a humidified atmosphere at 37 °C and 10% CO_2_. No antibiotics were used during routine subdivisions or for cell culture experiments to avoid altering cell metabolism. Fibroblasts for use in experiments were harvested at 70% confluence, detached using trypsin-EDTA, counted using a haemocytometer; diluted and suspended at a concentration of 1 × 10^4^ cells/mL. 

#### 3.3.5. Assessment of Cell Viability (MTT Assay)

NIH-3T3 cells (200 μL) were seeded at a density of 1 × 10^4^ cells/mL in 96-well plates (CoStar, Corning, Canada). Sterile tissue culture water only was used as a negative control and culture media plus cells plus sterile tissue culture water used as a positive control. Serial dilutions at concentrations of 25, 50 and 100% were performed on all specimens. Briefly, a 96-well plate was incubated for 24 h in a cell culture incubator at 37 °C (10% CO_2_/95% air atmosphere), upon which the relevant concentrations of sterile tissue culture water and specimen extracts were added to appropriate wells for testing and incubation for 24 h (37 °C (10% CO_2_/95% air atmosphere)). Analysis of each specimen extract was performed in triplicate (n = 3 extracts per condition), with 3 cell viability analyses performed on each extract. Each well was exposed to 5 mg/mL of MTT reagent (M2128, Sigma Aldrich, Oakville, ON, Canada) at an amount equal to 10% of the culture media volume before being returned to the incubator for a further 3 h. Post incubation, MTT solubilization solution (Catalog Code: M8910) was added at a volume equal to the original culture media volume. Absorbance of each well was spectrophotometrically measured at a wavelength of 570 nm on a multidetection microplate reader (Synergy HT, BIO-TEK, Winooski, VT, USA). Cell positive control wells were assumed to have 100% metabolic activity corresponding to cellular viability of 100% and the percentage cell viability of the cells exposed to experimental extracts were calculated relative to this.

### 3.4. Host Response Evaluation

#### 3.4.1. Circular Dichroism Spectropolarimetry

To investigate unwanted changes in Fg conformation onto the surface of the dissolution by-products, undiluted extracts (at 12, 24, 48, 96 and 120 h) of the experimental materials were analyzed using far-ultraviolet (UV) Circular Dichroism (CD) spectra, recorded using a Jasco J-810 spectropolarimeter (Easton, MD, USA) with temperature control capability. A 6.4 mg/mL solution of fibrinogen from human plasma (F4883, plasminogen free, containing 58% protein, 96% clottable protein; Sigma Aldrich, Oakville, ON, Canada) in 25 mM sodium phosphate buffer adjusted to pH 7.4 ± 0.05 using NaOH and H_2_SO_4_ was diluted to a concentration of 0.2 mg/mL with the solutions of embolic extracts (containing the ion dissolution products, as prepared in Section 3.3.1 (ORP1-3, ORP5-7, ORP9, ORP11 at n = 3 for each extract)). The concentration of the stock Fg solution was determined by UV spectroscopy at 280 nm using the manufacturer’s molar extinction coefficient. All spectra were collected at 37 °C (controlled with a NESLAB RTE-111 bath, Thermo Scientific, Newington, NH) in three repetitions (260–190 nm, 1 nm steps, 50 nm/min) in a 0.5 mm pathlength quartz cuvette (Hellma, Mülheim, Germany). The triplicate spectra for each ion dissolution solution were averaged, blank subtracted with phosphate buffer (25 mM; pH 7.4 ± 0.05), and converted to mean residue ellipticity ([θ]). Relative fibrinogen conformation between samples was monitored by comparing [θ] at the minima corresponding to the α-helical bands at ~208 and 222 nm [27]. 

#### 3.4.2. Platelet Lactate Dehydrogenase (LDH) Activity

The adhesion of platelets on the materials were confirmed based on the determination of lactate dehydrogenase (LDH) activity, to investigate the experimental materials potential to capture and activate platelets versus contour PVA, inducing site-specific thrombus formation, while limiting the blood supply to the target area without inducing a generalized or systemic pro-thrombotic state. All protocols pertaining to the use of whole blood and platelets were approved by the Capital Health Research Ethics Board according to protocol number: CDHA-RS/2012-003. The blood (31.5 mL) was collected *via* venipuncture from six healthy, aspirin-free human volunteers at the Laboratory Blood Collection facility at the Victoria General Hospital in seven 4.5 mL glass BD Vacutainer tubes (Catalog No. 364606, Becton-Dickinson, Franklin Lakes, NJ, USA) containing an acid-citrated dextrose (ACD) anticoagulant. It is important to note that the first tube (4.5 mL) of blood was discarded, as it is rich in clotting factors, and then the remaining 27 mL was collected. Platelet rich plasma (PRP) was generated by centrifuging the ACD-anticoagulated blood (1500 rpm, 8 min, 25 °C) using an Eppendorf 5702 centrifuge. Careful transfer of the PRP to individual centrifuge tubes was completed using sterile plastic pasteur pipettes. Platelet concentration was measured using a LH 785 CBC analyzer. The platelet concentration was recorded for each patient but not adjusted. The platelet suspension was then added to the preferred experimental embolic particle (ORP5 [19]), using contour PVA as the commercial control. A final concentration of 0.1 cc embolic material per mL of platelet suspension (3 mL of patient PRP for each embolic particle with the exception of one patient (No. 1006) which only had enough PRP to add 2.5 mL of PRP per embolic particle) was used and allowed to adhere for 1 h at 37 °C under static conditions. Subsequently, the suspension was aspirated from each well, and the non-adherent platelets were rinsed away by filling and aspirating the wells ten times with 2.5 mL of PBS. The entire duration from blood collection to the conclusion of the platelet adhesion step was less than 4 h. The adhesion of platelets on the materials surface were confirmed based on the determination of LDH activity, and analyzed at the clinical hematology laboratory at Capital Health (5788 University Ave, Mackenzie Building, Halifax, NS, Canada). Briefly, ORP5 (experimental embolic particle) and contour PVA (commercial embolic particle control) immersed in PRP solution, were washed two times with phosphate buffered saline (PBS) and lysed with 1% Triton-X-100. The absorbance was read at 340 nm on the UniCel DxC 800 Synchron^®^ automated analyzer (Becton-Dickinson, Franklin Lakes, NJ using the Synchron kit (Becton-Dickinson, Franklin Lakes, NJ, USA). A calibration curve was used to relate the platelet number to LDH activity.

### 3.5. Statistical Analysis

Each experiment was performed in triplicate and analyzed using Prism 5.0 software (Graphpad software Inc., La Jolla, CA, USA) Results are expressed as mean ± standard deviation of the triplicate determinations. For cytotoxicity, one way analysis of variance (ANOVA) was carried out followed by a Newman-Keuls post hoc test for comparisons between groups. The level of significance was set at *p* < 0.005 and *p* < 0.05.

## 4. Conclusions

The use of glass based materials offers a unique approach to the challenge of offering clinicians intrinsically radiopaque materials for embolization procedures. In line with the emerging philosophy of having dissolution by-products establish key interactions with living responses, we have developed a unique series of experimental embolic particles that may facilitate key material and host responses without the induction of any cell damage, similar to the commercially available contour PVA. We have successfully prepared a glass-based embolic particle with controlled levels of dissolution in terms of their leached by-products to offer either equivalent or superior levels of cytotoxicity and haemocompatibility to contour PVA. The platelet response testing evaluated herein promotes the requirement for further haemocompatibility testing (*complement activation, thrombosis* (*through evaluation of percentage occlusion in an animal model*)* and haemolysis*). Overall, the *in vitro* studies demonstrate promising early material and host (safety) responses for these novel glass-based particles, to substantiate their use in a pre-clinical evaluation.
